# Lipid Pocket Binders Impose Allosteric Changes of Protein Dynamics Around the Active Site of the Protein Kinase p38α

**DOI:** 10.1002/anie.202522665

**Published:** 2026-03-01

**Authors:** Sara Medina Gómez, Laurin T. Homberg, Mike Bührmann, Daniel Rauh, Rasmus Linser

**Affiliations:** ^1^ Department of Chemistry and Chemical Biology TU Dortmund University Dortmund Germany; ^2^ Department of Chemistry and Chemical Biology and Drug Discovery Hub Dortmund (DDHD) am Zentrum für Integrierte Wirkstoffforschung (ZIW) TU Dortmund University Dortmund Germany

**Keywords:** allosteric inhibitors, drug discovery, medicinal chemistry, NMR spectroscopy

## Abstract

Protein kinases represent major pharmaceutical targets, but the development of selective modulators remains challenging. In search of allosteric sites in the serine/threonine kinase p38α, a “lipid pocket” in the C‐lobe has been found to bear prospects for the binding of small molecules. A pharmacological potential of those low‐affinity binders found initially has not become obvious, however, raising the overarching question whether any sort of communication between this pocket and the enzyme's functional sites exists. Here, we use NMR spectroscopy to reveal an effective connectivity of these sites in spite of their spatial distance. The data reveal a clear interdependency of protein dynamics between the different structural elements through dynamic allostery, together suggesting a pharmacological avenue for the development of suitable lipid pocket binders to allosterically alter enzymatic functionality in a disease context.

## Graphical abstract

Used in search of novel allosteric sites in the serine/threonine kinase p38α, NMR spectroscopy reveals a dynamic coupling between the catalytic and lipid pockets. The findings uncover a motional interdependence that links distant regions of the enzyme, highlighting the lipid pocket as a promising site for allosteric intervention.

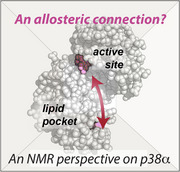

## 1 Introduction

Allosteric regulation describes the possibility for protein function to be modulated by binding events distant from the site responsible for catalytic activity or distant from interaction sites responsible for initiation of downstream regulatory cues [1, 2, 3]. Such mechanisms are indispensable in the cell to grant selectivity despite a high degree of conservation of surface features (e.g., for active sites in kinases and phosphatases, among others) across protein families [4, 5, 6, 7]. On the other hand, allosteric modulation has also been exploited as a pharmacological avenue to specifically address a wide range of drug targets [8, 9, 10]. Opposed to small molecules that bind to the active site (orthosteric inhibitors), allosteric binding can either increase or decrease activities, leading to a large spectrum of potential applications with a high specificity.

Limiting the important benefits that allosteric modulation is arguably associated with, identification of sites in a protein that are both druggable (specifically, that allow high‐affinity association of synthetic small molecules) and bear a sizable effect on enzymatic activity or on the affinity to third‐party interaction partners has constituted a major challenge [11, 12]. Allosteric pockets can be untraceable when unoccupied and form only in the presence of a suitable binder [10]. Understanding the underlying biophysical coupling between allosteric and active site has hence been associated with a growing scientific interest, in natural regulation and pharmacology [13, 14], but also for other applications like, for example, designer epigenetic readers [15, 16] or improved catalytic performance in a biotechnological context [17, 18].

Whereas allosteric regulation has often been associated with an interplay of distinct conformational states involving substantial structural rearrangements [19], modulation of protein dynamics has been suggested to play a similarly effective role to affect protein function [4, 20]. This form of “dynamic allostery” relies on networks of sequential interactions that can propagate through the protein scaffold over longer distances. In this case, ligand binding to one site can globally alter the molecular partition functions of the system (i.e., the distribution of motional modes) and hence thermodynamic (mean free energies and equilibria) and kinetic properties (feasibility of activation steps) even at distant sites [20]. The mechanistic details regarding the mutual influence of site‐specific motions and their consequences have received much scientific interest, embracing experimental work, simulations, and combinations thereof [5, 21, 22, 23].

**FIGURE 1:**
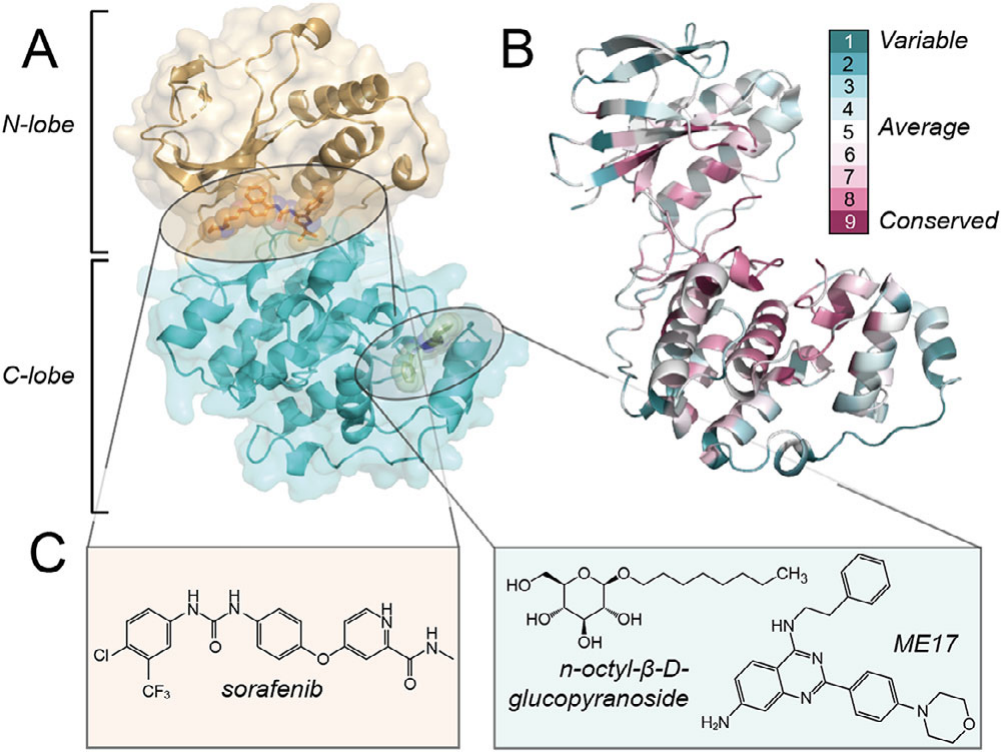
Topology of the protein kinase p38α and its ligand‐binding pockets. (A) Structure of p38α kinase highlighting ligand‐binding sites near the active site (orange) and the lipid pocket (green), based on PDB coordinates 1WFC (p38α), 1KV2 (active‐site inhibitor), and 5N68 (lipid pocket inhibitor) [43]. (B) Sequence conservation of the primary structure [44] with conserved residues being represented in purple (see color code to its right and compare Figure S1). (C) Chemical structures of the orthosteric inhibitor sorafenib (left) and the lipid pocket ligands n‐octyl‐*β*‐D‐glucopyranoside and the quinazoline ME17 (right).

The family of protein kinases represents ubiquitously important drug targets [24]. However, just like phosphatases and several other enzyme classes, they bear a high structural similarity between the active sites across the entire protein family. This has rendered their selective targeting a major challenge, frequently associated with off‐target effects [25]. Kinase regulation is thought to involve a rich spectrum of conformational dynamics that underpin their functionality [26]. This includes the presence of structural “spines”, which allow a twisting of the lobes relative to each other [21]. Moreover, the importance of allostery for their activation, for example, by dimerization [27, 28] or interactions with SH2 domains stabilizing the regulatory αC helix [29, 30], is well known. Communication between the catalytic and substrate‐binding sites is thought to involve widespread dynamic networks [21, 31, 32, 33]. Interestingly, this mutual dynamic connectivity seems to include both, the N‐ and the C‐lobe of kinases (see Figure 1A for a structural representation of p38α kinase) [21, 32, 33, 34, 35]. This is consistent with the high degree of primary‐sequence conservation of the C‐lobe, despite much of it not being directly involved in the enzymatic process (see p38α sequence conservation in Figures 1B and S1) [36]. The serine/threonine kinase p38α, a member of the mitogen‐activated protein kinase (MAPK) pathway, is a well‐established drug target due to its prominent role in inflammation [37, 38, 39, 40]. Likewise, with its relatively low molecular weight and complexity, it has been used as a prototype protein kinase from both, structural and biophysical viewpoints [41, 42].

In previous work, a large second pocket has been identified in the C‐lobe, distant from the active site. This partly conserved “lipid pocket”, whose “entrance” corresponds to the F‐motif recruitment site (FRS) or DEF pocket, which binds F(xFP)‐type motifs [45], has the potential to open and accommodate lipids as well as drug‐like small molecules (Figures 1C and S2). This has been shown conceptually via protein X‐ray crystallography of complexes involving non‐covalent or covalently tethered ligands (lipid pocket ligands, “LiPoLis”, Figure 1C) [40, 43, 46]. As expected, the crystallographic structures obtained for apo p38α (PDB 1WFC) and for p38α in the presence of the lipid pocket ligands n‐octyl‐β‐D‐glucopyranoside (also called β‐octylglycoside or BOG, PDB 3MH3) [47, 48] and 2‐(4‐morpholin‐4‐ylphenyl)‐∼{N}4‐(2‐phenyl)quinazoline‐4,7‐diamine (henceforth called ME17, PDB 5N68) [43], as well as those for the kinase bound to the active‐site inhibitor sorafenib, either in additional presence or absence of BOG (PDB 3GCS and 3HEG, respectively) [49, 50] are nearly identical. However, site‐specific comparison of (normalized) temperature B‐factors (compare Figure S3) reveals that, apart from an impact directly at the lipid pocket, other elements of the scaffold also experience subtle changes in thermal vibration. Hence, lipid pocket occupation could potentially have an impact on the prevailing dynamic conformational equilibrium in the sense of “dynamic allostery” [20]. At this point, however, it remains unclear whether the lipid pocket, positioned at a distance of 35 Å from the active site and separated by the cleft between the N‐ and the C‐lobe, is motionally connected to the active site or not. Plainly, in the words of Cooper and Dryden [20], the question is whether atoms in one site “in some way feel the presence of a ligand” at the other site. This knowledge would have a substantial impact on the usefulness of future studies that aim to exploit the site pharmacologically. For example, whereas the affinity of the compounds synthesized to bind to it in a noncovalent way has so far remained rather low (down to the µM range) [43], a chemical‐genetic approach has demonstrated that tailored covalent ligands are suitable to achieve effective targeting of the binding site—although, as of now, in an artificial setting [40]. Likewise, the identification of sufficiently bulky binders might111 enhance a detrimental (constitutively down‐regulating), long‐range impact on active‐site integrity, kinase activation kinetics, or substrate binding competency. Meaningful downstream investment in medicinal chemistry, biochemical characterization, and structural biology is only justified if newly identified allosteric pockets are functionally linked to sites that control protein activity and regulation. Here, we utilized solution NMR spectroscopy to interrogate the interdependency of site‐specific motion at the lipid pocket and around the active site. The data reveal an unambiguous connectivity between motional parameters at the active site and lipid pocket occupancy, which may serve as a general basis for more complex medicinal‐chemistry work on allosteric modulation of kinases using C‐lobe‐binding compounds.

## 2 Results and Discussion

Triply labeled [^2^H, ^15^N, ^13^C] p38α was expressed in M9 minimal media using a deuteration‐adapted protocol and purified following established procedures (See details in the Supporting Information). Backbone resonance assignment was achieved on the basis of a standard suite of 3D sequential backbone experiments, aided by published chemical shifts from past work [41, 51] obtained under similar experimental conditions (BMRB entry 17471). To better understand how a possible occupancy of the lipid pocket affects the enzyme's overall properties, we involved two known lipid pocket binders and compared the bound states with the enzyme in the absence of said compounds. To quantitatively interrogate ligand‐induced changes in the conformational ensemble, we adopted a consistent workflow in which we systematically probed (i) changes in the chemical shifts of each backbone amide moiety upon ligand binding (chemical‐shift perturbations, CSPs), (ii) changes in the Redfield relaxation parameters *R*_1_, *R*_2_, and the heteronuclear ({^1^H}^15^N steady‐state) nuclear Overhauser effect (hetNOE), and (iii) changes in Carr‐Purcell‐Meiboom‐Gill (CPMG) Bloch‐McConnell relaxation dispersion profiles. CSPs report on changes in the average local chemical environment (e.g., bond angle/length distributions, vicinity to aromatic groups, etc.) on timescales up the ms regime. Changes in *R*_1_ and hetNOE reflect differences in fast (ps‐ns) timescale dynamics of the amide bond vector, whereas *R*_2_ rates, obtained with a 2 kHz CPMG pulse train to limit contributions from ms‐timescale dynamics, are sensitive to both, fast and intermediate (µs) timescale dynamics. In addition, the exchange contribution (*R*_ex_) extracted from relaxation dispersion data reports on µs‐ms‐timescale conformational exchange.

**FIGURE 2:**
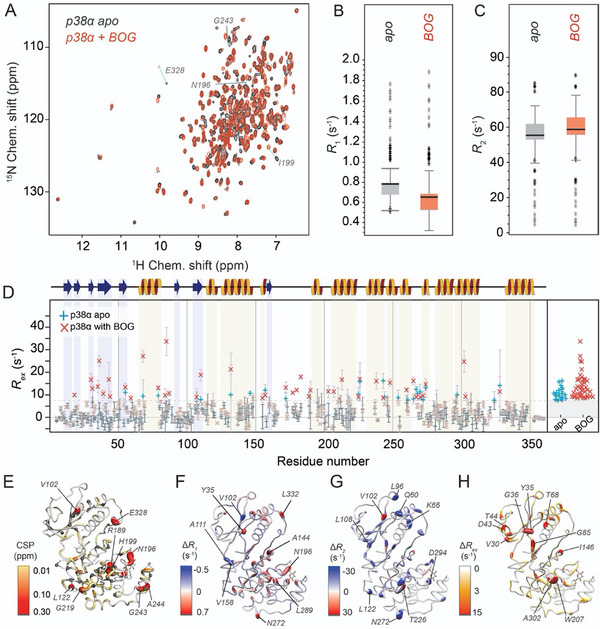
Impact of n‐octyl‐β‐D‐glucopyranoside (BOG) binding to p38 α. (A) Overlay of ^1^H‐^15^N TROSY spectra of apo (black) and BOG‐bound p38α. (B) *R*_1_ relaxation rates for apo and BOG‐bound p38α, represented as a box plot. (C) *R*_2_ rates found for apo and BOG‐bound p38α. (D) Residue‐specific *R*_ex_ values from CPMG relaxation dispersion experiments as a function of sequence, highlighting those residues that bear contributions larger than 7.5 s^−1^ (bright blue and red crosses for apo and BOG‐bound protein, respectively, with a distribution of those values shown on the right). Values below this threshold are included only in the left part using faint colors. (E) Chemical‐shift perturbations (computed as [(Δ(δ^1^H)^2^ + Δ(δ^15^N)^2^/10]^1/2^), mapped onto the p38α structure (PDB entry: 3MH3). Residues disappearing when BOG is present are depicted as black spheres. (F) Difference in *R*_1_ rates, represented by color and sphere size (then as their magnitude), between apo and BOG‐bound p38α (apo minus BOG‐bound). (G) Differences between *R*_2_ rates for apo and BOG‐bound p38α as in F. (H) Differences in *R*_ex_ between apo and BOG‐bound p38α, represented in magnitude mode on the protein structure (PDB entry: 3MH3). Errors in D) correspond to uncertainties in *R_ex_* by standard Gaussian error propagation, starting from the experimental uncertainties of peak intensities used to derive *R_2_^eff^* values in the CPMG relaxation dispersion analysis.

We first pursued these comparisons for saturating concentrations of BOG, which is known to bind to the lipid pocket with a *K*_D_ of 3 µM [52] (see Figure 2A). Rather unsurprisingly, CSPs were found in the vicinity of the lipid pocket. Intriguingly, however, a considerable impact was also seen for part of the N‐lobe (e.g., 0.12 ppm for V102, a known reporter of long‐range conformational coupling, or 0.32 ppm for E328, linked to the αC helix, see Figure 2A, E).

In addition to these CSPs, some residues, such as A51, K53, or L86 from the ATP‐binding site, experience line broadening to the point of peak disappearance. Figure 2E depicts these perturbations on the structure, representing both, the magnitude of the CSPs as well as those residues becoming “invisible” in BOG‐bound p38α.

In the light of a possible “dynamic allostery” across the kinase structure, the above‐mentioned site‐specific dynamic properties of the kinase are of particular interest. Figure 2B,C (also compare Figures S4 and S5) display the overall distribution of *R*_1_ and *R*_2_ rates, respectively, of the protein with or without BOG bound. Whereas the longitudinal *R*_1_ rates in the BOG‐bound form are slightly lower than in the protein with an empty lipid pocket (Figure 2B), the overall *R*_2_ distributions are shifted towards higher values in the presence of BOG, including, but not limited to, the active‐site residues (Figure 2C). Differences between apo and BOG‐bound forms are visualized on the protein surface for *R*_1_ and *R*_2_ in Figure 2F,G, respectively. In addition to the moderate change regarding the overall distribution of *R*_1_ rates, the surface representation in Figure 2F reveals that individual residues change very strongly (up to 0.5 s^−1^). The most prominent differences occur near the lipid pocket (see e.g., N196 and L289); however, it is noteworthy that differences are also observed for individual residues in the N‐lobe (e.g., Y35, I63, A144, and L332). All of these residues show reduced *R*_1_ rates in the BOG‐bound form, consistent with reduced flexibility in the N‐lobe. By contrast, several residues close to the hinge region and within the N‐lobe (e.g., V102, A111, and V158) display increased *R_1_* rates upon BOG binding, indicating increased flexibility at these specific positions. Differences in dynamics upon BOG addition, extending well beyond the pocket, are even more apparent from the *R*_2_ rates (Figure 2G), with changes exceeding 20 s^−1^ (e.g., Q60, K66, L96, L122, T226, N272, and D294). Many of these residues, including the functionally relevant hinge region (residues 106 to 113), glycine‐rich loop (31‐36), and the N‐lobe (e.g., Q60 and K66) generally, show a widespread, pronounced increase, indicating a tendency towards a stiffer architecture. Notably, lower rates for individual residues are also observed, exemplified by V345 in the C‐terminal helix αL16 or the adjacent V102. Additional analyses employing {^1^H}‐^15^N heteronuclear NOE measurements confirm the dynamic alterations, however, with changes not as pronounced as for *R*_1_ and *R*_2_ (Figures S6 and S7).

Finally, the CPMG relaxation dispersion experiments allowed us to compare the extent and distribution of conformational exchange between the apo and BOG‐bound form of p38α. Figure 2D visualizes the sites that are characterized by strong (*R*_ex_ > 7.5 s^−1^) exchange contributions for apo and BOG‐bound p38α as a function of residue. While conformational exchange is detectable in both forms, the BOG‐bound protein exhibits more than twice as many residues with strong exchange contributions, and the magnitude of *R*_ex_ is markedly increased compared to the apo state, exceeding 2‐fold at several positions (e.g., G85 in the ATP‐binding site, G36, T68, and A302). Strikingly, this increase in exchange is not confined to the lipid pocket but again extends to distal regions of the protein. Structural mapping of *R*_ex_ differences between apo and BOG‐bound forms onto the p38α crystal structure (Figure 2H) highlights the widespread nature of these dynamic changes. The difference in *R*_ex_ between the two forms aligns well with the trends observed in the *R*_2_ rates, reinforcing the conclusion that BOG binding induces global alterations in conformational dynamics across the entire protein scaffold, in particular also including the N‐lobe and the active site.

**FIGURE 3:**
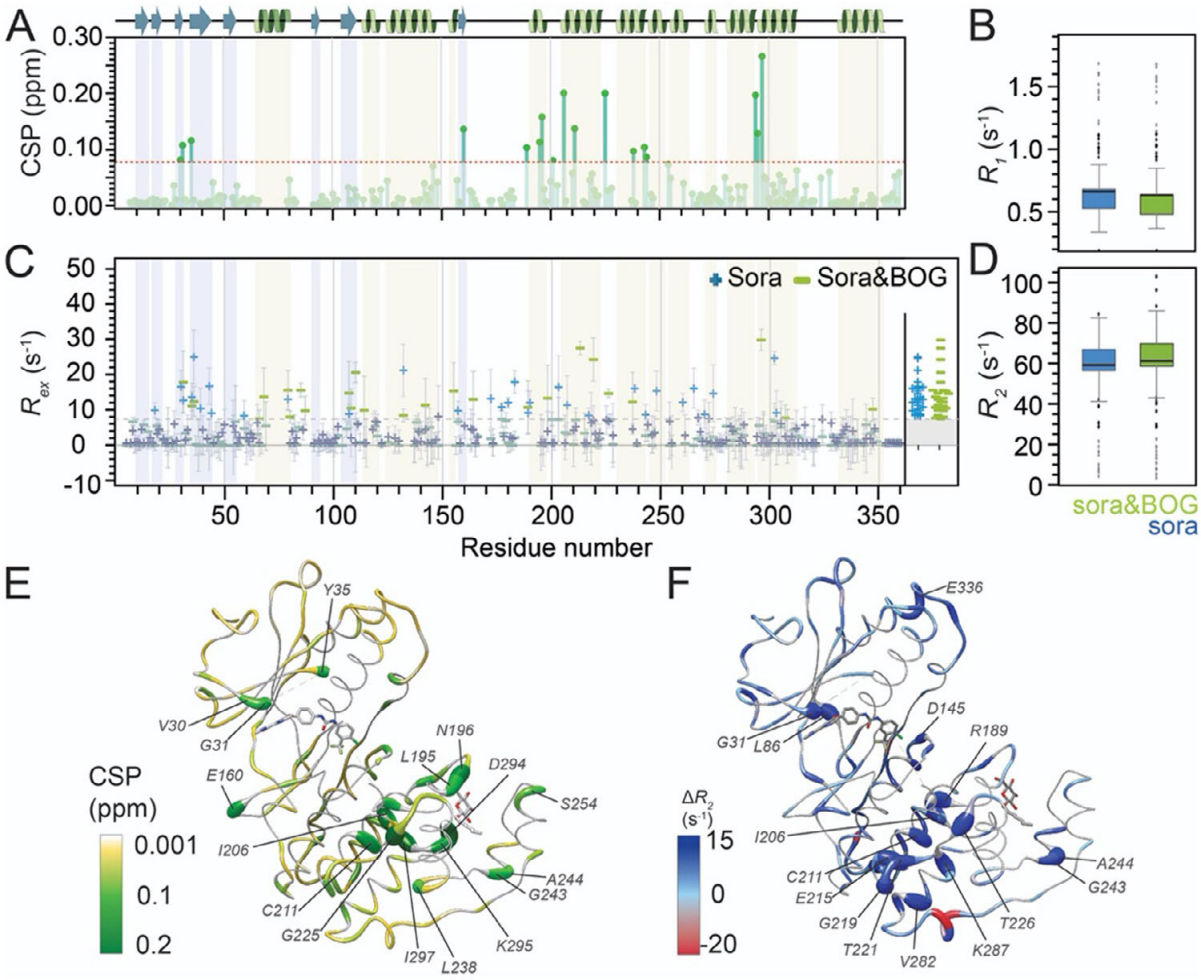
Impact of lipid pocket occupancy on protein motion in the presence of the orthosteric inhibitor sorafenib. (A) Chemical‐shift perturbation upon BOG binding onto sorafenib‐bound p38α. (B) Changes in *R*_1_ distributions upon BOG association onto sorafenib‐bound p38α. (C) Comparison of exchange contributions in sorafenib‐only‐bound p38α compared to p38α in the presence of both, sorafenib and BOG. Residues with significant conformational‐exchange contribution (*R*_ex_ > 7.5 s^−1^) are highlighted (lighter colors and right side). (D) Changes in *R*_2_ distributions. (E) Chemical‐shift perturbations upon addition of BOG to sorafenib‐bound p38α (PDB entry: 3GCS). (F) Differences in *R*_2_ rates between sorafenib‐bound and sorafenib‐ and BOG‐bound p38α (PDB entry: 3GCS). Errors in C) correspond to uncertainties in *R_ex_* by standard Gaussian error propagation, starting from the experimental uncertainties of peak intensities used to derive *R_2_^eff^* values in the CPMG relaxation dispersion analysis.

To investigate the influence of *orthosteric* modulation on the dynamic effects brought upon by lipid pocket occupancy, we prepared samples of p38α bound either to the Type II active‐site inhibitor sorafenib alone [53], or to both, sorafenib and BOG, and again pursued the comparative assessment outlined above. The CSPs observed upon BOG addition within the sorafenib‐bound background are reasonably consistent with the comparison shown in the absence of sorafenib (Figure 2A,E), suggesting that lipid pocket occupancy still alters the dynamic ensemble to some extent, despite the overall rigidifying effect of orthosteric modulation. Closer inspection of the CSP data reveals significant perturbations exceeding two standard deviations (0.08 ppm) for several residues in the C‐lobe that are either part of, or located near, the lipid pocket (Figure 3A,E, see e.g., I206, G225, D294, or I297). In addition, a small number of residues in the N/C‐lobe interface (e.g., D160, known from docking interactions with MAP kinase activated protein kinases) and the N‐lobe again display considerable perturbations also, particularly those adjacent to the glycine‐rich loop (V30, 0.08 ppm, and G31, 0.11 ppm) and near the ATP‐binding site (such as Y35, 0.12 ppm). Conversely, in terms of protein dynamics, the comparison between sorafenib‐only and sorafenib plus BOG‐bound states reveals trends similar to those observed in the absence of sorafenib, although to a reduced extent. Specifically, BOG binding leads to a subtle but discernible decrease in *R*_1_ and increase of *R*_2_ rates (Figure 3B,D), consistent with an overall but weaker reduction of ps–ns timescale dynamics again. This trend is most prominent for residues adjacent to the lipid pocket (e.g., R189, T226, and K287), but is also observed for a few sites in the glycine‐rich loop (G31), near the active site (L86, and D145), and the C‐terminal helix αL16 (E336). The increase of motion in the intermediate‐exchange regime upon lipid pocket occupation (as witnessed by *R*_ex_ contributions, Figure 3C), however, is barely visible. Hence, while the presence of the orthosteric inhibitor prevents  larger‐scale conformational exchange, as seen elsewhere [54, 55, 56], on the fast timescale, lipid pocket occupancy still seems to induce localized entropic changes that extend across the protein structure.

**FIGURE 4:**
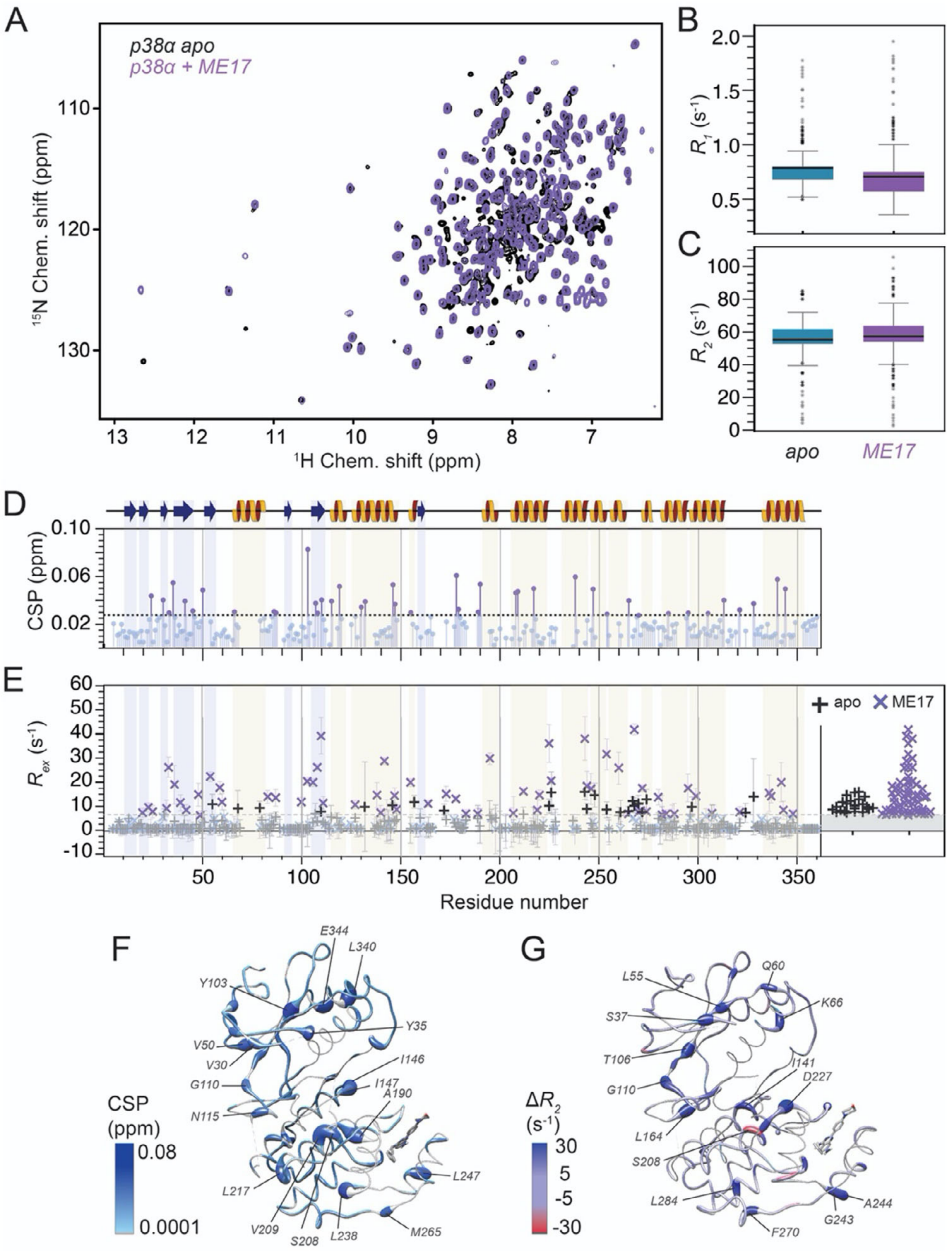
Impact of the drug‐like lipid pocket binder ME17. (A) Overlay of H/N TROSY spectra of apo p38α (black) and p38α saturated with ME17 (purple). (B) Changes in *R*_1_ distributions upon ME17 association. (C) Changes in *R*_2_ distributions upon ME17 association. (D) Chemical‐shift perturbations upon ME17 association. The dashed line denotes residues with the strongest CSPs (two times the standard deviation). (E) Comparison of exchange contributions in apo (black) and ME17‐bound p38α (purple). The right side highlights the distribution of values exceeding 7.5 s^−1^. (F) Chemical‐shift perturbations upon ME17 association into apo p38α mapped onto the crystal structure (PDB entry: 5N68). (G) Changes in *R*_2_ rates upon ME17 association (PDB entry: 5N68). Errors in C) correspond to uncertainties in *R_ex_* by standard Gaussian error propagation, starting from the experimental uncertainties of peak intensities used to derive *R_2_^eff^* values in the CPMG relaxation dispersion analysis.

BOG represents a rather simple model compound binding into the lipid pocket. To test whether the effects we observed above would also apply to more drug‐like, noncovalent lipid pocket binders, we performed the comparative assessment outlined initially for the small‐molecule compound ME17 (Figure 4), which has a K_D_ of ∼240 µM [43]. The resulting CSPs, compared to apo p38α (Figure 4A,D), are qualitatively similar to the differences between BOG‐bound and apo forms. Perturbations occur throughout the protein, with significant changes near the lipid pocket (e.g., A190 or L247), as expected, but also inducing notable shifts in the N‐lobe (V50 and Y103) and several residues in the C‐terminal helix αL16 (e.g., E340, L344). Residues exceeding the two‐standard‐deviation threshold are distributed across both lobes, suggesting a more delocalized, but weaker, effect compared to BOG (Figure 4D,F). As observed for BOG in the absence or presence of active‐site occupancy, the *R*_1_ rates for ME17 were found to be systematically lower in comparison to apo p38α (Figure 4B), while the *R*_2_ rates are comparable but tend to be marginally higher (Figure 4C). Mapping the *R*_2_ differences (apo minus ME17) onto the structure (Figure 4G) highlights positive values of up to 30 s^−1^. This includes residues near the lipid pocket, such as G243 and A244, but also sites at/adjacent to the functionally important αC helix (K66), glycine‐rich loop (S37, L55), and hinge region (T106, G110), as well as other parts of the N‐lobe (L55, Q60). Similar to BOG, the exchange contributions *R*_ex_ in the presence of ME17 are again slightly increased compared to the apo protein (Figure 4E). Relative to the lower extent of CSPs, the exchange contributions here, extending again far into the N‐lobe, even seem more pronounced than those induced by BOG. This may be due to faster association/dissociation compared to the higher‐affinity BOG, potentially reaching the µs timescale regime and adding/spreading conformational exchange motion throughout the enzyme here. Together, these observations indicate that ME17, despite its distinct chemical nature and drug‐like character, induces a dynamic response qualitatively similar to that of BOG, albeit of smaller amplitude and with a broader spatial distribution of affected residues.

The lipid pocket in p38α bears a substantial distance and relative structural separation from the N‐lobe of the kinase domain. Nevertheless, the above data across multiple observables consistently suggest that ligand binding to the lipid pocket also affects the residues in the N‐lobe. These effects encompass chemical shifts, which act as scalar representations for the multi‐dimensional parameter space of the time‐dependent conformational ensemble, as well as different types of site‐specific ^15^N relaxation, which collectively suggest a sizeable motional interdependency between the lipid pocket and the N‐lobe. Generally, the presence of chemical‐shift perturbations can derive from motional differences, structural differences, or both. Lipid pocket occupation has previously incurred slight changes in cryogenic B‐factors that extend from the lipid‐binding domain to the active site and beyond (Figure S3) [40, 43]. The relaxation data recorded at room temperature in solution, both regarding fast (ps–ns) as well as slower (µs–ms) time scales, confirm the observed impact to derive from changes in the motional characteristics within both lobes.

**FIGURE 5:**
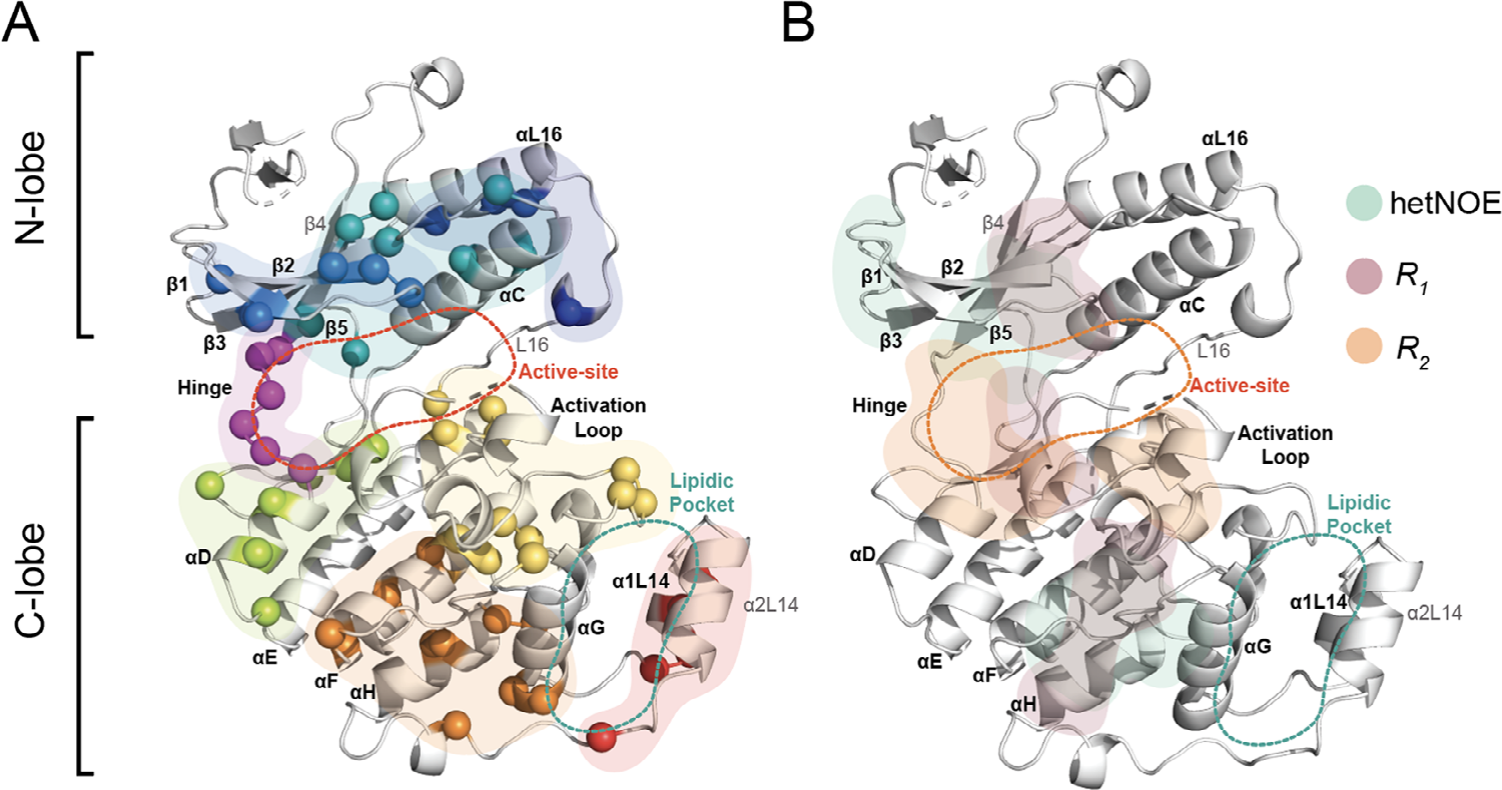
Structural clustering of residues exhibiting ligand‐induced dynamic and chemical‐shift changes in p38α (PDB entry: 1WFC). (A) X‐ray structure of apo p38α highlighting structural elements that display significant differences between the apo protein and BOG‐ or ME17‐bound states, as identified from CSPs and CPMG relaxation dispersion, reflecting intermediate‐timescale dynamic effects. Individual residues with significant changes, that is, exceeding two standard deviations relative to the experimental distributions, are shown as colored spheres according to the (secondary‐) structural elements they belong to. (B) Clusters derived from differences in Redfield‐type relaxation parameters between apo and different ligand‐bound states, shown separately for hetNOE (green), *R_1_* (red) and *R_2_* (orange). The identities of the individual residues comprising each cluster are provided in Figure S8.

A more consolidated spatial analysis of sites exhibiting significant ligand‐induced differences (defined as exceeding two standard deviations relative to the experimental distribution) further refines this view (Figure 5). Residues affected across CSPs, CPMG, steady‐state {^1^H}‐^15^N heteronuclear NOE, *R_1_*, and *R_2_* measurements were grouped into clusters based on their spatial proximity. The depiction of chemical‐shift perturbations and conformational‐exchange contributions (Figure 5A) shows that residues are affected very widely across the various secondary‐structural elements of the enzyme, with the exception of the N‐terminal 20 residues, the loop L6 (residues 90–100) and the αE helix, making it difficult to pinpoint one specific main localization of the impact that lipid pocket occupation has. Fast‐timescale dynamic changes, reflected primarily in *R*_1_ and heteronuclear NOE data, however, mostly affect residues located at the end of β‐strands β2 and β3, loops L1 and L2, and helices αF and αG. These changes likely denote a global reorchestration of statistical thermodynamics across the tightly interconnected fold and might denote a rather secondary effect rather than being on‐pathway. By contrast, signatures of intermediate‐timescale dynamics, captured by differences in CSPs, CPMG‐derived *R_ex_*, and *R_2_*, even though still distributed broadly, most prominently involve the functionally important hinge region and helix αC as well as Loop L12 (Figures 5B and S8). This observation is consistent with the involvement of more central elements in between the N‐and the C‐lobe, which are known to be reasonably plastic and to fulfill functions associated with spatial reorganization. We wondered whether changes on the intermediate timescale might be associated with higher evolutionary conservation on the residue level, but the arising picture (see a qualitative comparison in Figure S9) is insufficiently clear to draw any significant conclusions.

The apparent motional interdependencies involving the lipid pocket is in coherence with the general picture of widespread connectivity between catalytic, substrate‐binding, and regulatory sites that has been concluded from simulations for other kinases in the past. On a molecular level, the residues lying in the plastic interface seem to form a complex network of coupled oscillators. Changes in the statistical thermodynamics of the lipid‐binding domain hence translate into differential distributions of microstates of the structural elements in between the lobes, such as the activation loop, and above. Compared to the mentioned structural spines, however, which have been deduced from MD simulations to form upon activation of the kinase, our data denote a surprisingly broad involvement of individual residues in the interface between N‐ and C‐lobe, many of them of functional significance, into the network of motional dependencies. Unfortunately, those residues that are expected to bear the most pronounced dynamics, in particular the activation loop bearing the DFG motif, are not resonance‐assigned (probably exchange‐broadened/invisible) and hence escape all NMR analyses. We expect that these residues, prominently centered between the N‐ and C‐lobe and largely responsible for the intermolecular interaction underlying enzymatic function, play a major role in signal transduction particularly for motion on the intermediate timescale. NMR spectroscopy being an ensemble technique, residues affected by pocket occupation (and how) are potentially identified. But how their motion is correlated, and hence what residues represent the direct motional couplings and pathways, remains elusive. Similarly, the assessment here only includes changes at the backbone amides, whereas other (sidechain) atoms, such as hydrophobic contacts, might be more important to map the extent of motional connectivities and architecture of motional networks. In future work, with the limitation of being restricted to fast‐timescale motional couplings only, detailed assessments in silico, such as dynamic‐network analysis [57], together with proper experimental verification (e.g., by mutagenesis), may grant the specific paths of communication and pinpoint hotspots, including the residues that are invisible by NMR.

Importantly, even from this rather qualitative viewpoint, the confirmation that occupation of the lipid pocket does have an impact on the catalytic site and other functional regions encourages drug discovery and medicinal‐chemistry efforts directed to attain pharmacological lipid pocket binders. First‐generation ligands based on differently substituted quinazoline scaffolds could be characterized by protein x‐ray crystallography but failed to bind with sufficient affinity [43]. The above suggests, however, that the desired pharmacological potency might well be achieved involving either dedicated high‐throughput screening based on a greater variety of scaffolds, including nonplanar and more specifically shaped compounds, or more involved virtual screening attempts. Since it is not the mere presence (as, e.g., for orthosteric inhibitors, which mostly compete with substrates) but the effect on the dynamic network that is sought, a broad exploration of the available chemical space and generous variations of the bulkiness of the ligand seem to be useful avenues. Moreover, given the widespread connectivity of protein motion throughout both lobes as well as the rather plastic scaffold of the C‐lobe, and taking the lipid pocket as a guide, a specific search for other cryptic binding sites in the C‐lobe may bring further prospects to impact p38α function via dynamic allostery. In relatively small kinases as in p38α here, site‐specific motional properties appear to be extensively interconnected across the overall structure, and the major quest will be to find pockets that grant high‐enough affinity. Conversely, NMR interrogation of larger proteins, including by methyl‐based approaches, may specifically rule in or out motionally connected regions, which can facilitate, for example, dynamic‐docking‐based virtual screening.

## 3 Conclusions

In conclusion, we could show that the association of different molecules to the “lipid pocket” of the protein kinase p38α exerts a sizeable impact on the room temperature conformational ensemble, involving both, the catalytic site as well as the distal regulatory site of the kinase. Whereas previously, the prospects of targeting this pocket have remained questionable, the confirmation of dynamic interconnectivity with the active‐site architecture acts as a motivation for further drug discovery efforts aimed at exploiting it for the development of more potent allosteric modulators. The overall motional connectivity between the two lobes of p38α also highlights the potential to identify additional surface‐exposed sites with a likely regulatory effect in an allosteric manner generally if sufficient affinity can be achieved.

## Conflicts of Interest

The authors declare no conflicts of interest.

## Supporting information




**Supporting File 1**: The authors have cited additional references within the Supporting Information [58‐61].

## Data Availability

The data that support the findings of this study are available under doi.org/10.17877/TUDODATA‐2026‐JFINFJ.
