# Supplementary material for: Lipid Pocket Binders Impose Allosteric Changes of Protein Dynamics Around the Active Site of the Protein Kinase p38α
Source: Angew Chem Int Ed Engl. 2026 Mar 1;65(15):e22665. doi: 10.1002/anie.202522665 (PMC13053915; doi:10.1002/anie.202522665)
Supplement: Supplementary file 1 — Supporting File 1: The authors have cited additional references within the Supporting Information [58‐61]. [file ANIE-65-e22665-s001.pdf]

Supporting information for

Lipid Pocket Binders Impose Allosteric Changes of Protein Dynamics around the Active Site of the Protein Kinase p38α

Sara Medina Gómez[1], Laurin T. Homberg[1], Mike Bührmann[1], Daniel Rauh[1], Rasmus Linser[1]*

[1]Department of Chemistry and Chemical Biology, TU Dortmund University, Otto-Hahn-Str. 4a, 44227 Dortmund.

*To whom correspondence should be sent: rasmus.linser@tu-dortmund.de

Contents

Protein expression and purification ***1***

Solution NMR sample preparation ***2***

NMR spectroscopy (equipment, assignments, Redfield relaxation, CPMG) ***3***

Supplementary figures ***4***

Fig. S1: *Sequence conservation profile of p38α kinase* 4

Fig. S2: *Zoom-in to the lipidic pocket* 4

Fig S3: *Ligand-induced changes in crystallographic B-factors* 5

Fig. S4: *Comparison of ^{15}N longitudinal R_1 relaxation rates* 6

Fig. S5: *Comparison of ^{15}N transverse R_2 relaxation rates* 7

Fig. S6: *Distribution of heteronuclear NOE* 7

Fig. S7: *Comparison of steady-state $\{^1H\}$-^{15}N heteronuclear NOE values* 8

Fig. 8: *Mapping of residues exhibiting significant ligand-induced changes* 8

Fig. S9: *Sequence conservation and dynamic signatures of p38α* 9

Citations ***9***

Protein expression and purification

Chemically competent *E. coli* BL21(DE3) cells were transformed with the human p38α MAPK construct, cloned into a pNFG vector containing ampicillin and chloramphenicol resistance genes as well as a His_6-tag with thrombin cleavage site. Protein expression was induced at OD_{600} = 0.5 using 500 µM IPTG, followed by incubation at 18 °C for 20 h. For NMR experiments, cells were cultured in M9 minimal medium supplemented with 2 g/L $^2H_7{}^{13}C_6$ D-glucose, 1 g/L $^{15}NH_4Cl$ and D_2O. Bacteria cells were harvested by centrifugation at 4000 x g for 20 min, flash-frozen in liquid nitrogen, and stored at -80 °C. Pellets were resuspended in 3x volumes of lysis buffer (Buffer A), and 25 mg of lysozyme per 50 mL of sample and 20 µL Benzonase were added. After 15 minutes of incubation on ice, cell disruption was performed using a microfluidizer until the lysate was clear. The lysate was clarified by ultracentrifugation

at 110,000 x g for 45 min at 4 °C. Protein purification was initiated with affinity chromatography using a 5 mL HisTrap HP column pre-equilibrated with Buffer A. The column was washed with ten column volumes (CV) of Buffer A, and the bound proteins were eluted using a linear gradient of 0-100 % Buffer B over 20 CV at a flow rate of 5 mL/min. Protein fractions were analyzed by SDS-PAGE, and the major peak was pooled and dialyzed against 650 mL of dialysis buffer at 4 °C, with a 10 kDa molecular-weight cut-off (MWCO) and buffer changes every one to two hours over a total of four hours. Thrombin cleavage was performed by adding 300 µg/mL thrombin to the dialyzed sample, followed by overnight incubation at 4 °C. The cleaved sample was centrifuged at 4000 x g for 15 min, concentrated to 4-5 mL using a 10 kDa-MWCO concentrator, and diluted 5:1 in Buffer QA. The sample was then loaded onto a 5 mL HiTrap Q FF anion exchange column at the flow rate 5 mL/min. Non-bound proteins were washed out with 5 CV Buffer QA, and p38α eluted using a 0-100% Buffer QB linear gradient over 20 CV. Eluted fraction were analyzed by SDS-PAGE, and the main peak was pooled, concentrated to 2-4 mL, and subjected to size-exclusion chromatography (SEC) using a HiLoad16/600 Superdex 75 pg column at a flow rate of 1 mL/min. Fractions of 2 mL fractions were collected, and the main peak, corresponding to p38α, was confirmed by SDS-PAGE analysis and solution NMR.

Table S1. p38α purification buffers for affinity, anion exchange and size-exclusion chromatography and dialysis.

Buffer	Composition
A	50 mM Tris, 500 mM NaCl, 25 mM imidazole, 5% v/v glycerol, 1 mM DTT, 1 mM PMSF, pH 8
B	50 mM Tris, 500 mM NaCl, 500 mM imidazole, 5% v/v glycerol, pH 8
Cleavage/Dialysis	25 mM HEPES, 100 mM NaCl, 5% v/v glycerol, 1 mM DTT, pH 7
QA	25 mM HEPES, 5% v/v glycerol, 1 mM DTT, pH 7
QB	25 mM HEPES, 1M NaCl 5% v/v glycerol, pH 7
SEC	20 mM HEPES, 50 mM NaCl, 100 mg/L methionine, 5% v/v glycerol, 10 mM DTT, pH 7

Solution NMR sample preparation

After size-exclusion chromatography (SEC), protein samples were concentrated to a final concentration of ~7 mg/mL and divided into 2 mL aliquots. One aliquot was diluted with 10 mL of NMR buffer (50 mM HEPES, 150 mM NaCl, 5 mM DTT, pH 6.8) and subsequently reconcentrated to a volume of 1-2 mL. This buffer exchange was repeated with another 10 mL of NMR buffer, after which samples were concentrated to final desired concentration.

For the preparation of the p38α complex with BOG or ME17, the protein was first concentrated to the final desired concentration, then mixed with either a 75 mM stock solution of BOG in water (molar ratio of up to 1:4) or 50 mM stock solution of ME17 in DMSO (molar ratio of up to 1:6, protein:ligand). Chemical-shift perturbations were monitored out using 2D ^{15}N–^{1}H

TROSY-HSQC experiments by (over-)titrating either ligand into the protein. Data were analyzed using CCPNMR, and CSPs were computed using $[(\Delta\delta^{1}H)^{2} + (\Delta\delta^{15}N)^{2}/10]^{1/2}$.

For the preparation of p38α−sorafenib complex, the protein was concentrated to ~9.5 mg/mL, mixed with 50 mM sorafenib stock solution at a volume ratio of 40:1 (protein:ligand) and incubated on ice for one hour. Following incubation, the sample was centrifuged at 10,000 rpm for 2 min, and the resulting supernatant was concentrated to the final desired concentration. For the p38α−sorafenib-BOG complex, a 75 mM BOG stock solution was to the protein-sorafenib mixture (ratio 1:4) prior incubation on ice for 1 h; centrifugation and concentration were then carried out as described above.

NMR spectroscopy (equipment, assignments, Redfield relaxation, CPMG)

All NMR spectra were recorded at 293 K on a Bruker Avance NEO 800 MHz (^{1}H Larmor frequency) spectrometer equipped with a CP TCI proton-optimized triple resonance cryoprobe. For NMR assignment transfer the following experiments were recorded: 2D ^{15}N-^{1}H TROSY-HSQC, 3D-TROSY HNCA, and 3D TROSY-HNCACB.

^{15}N longitudinal (R_1) relaxation rates were acquired using [^{15}N,^{13}C,^{2}H]-labeled p38α in an interleaved manner, using a recycle delay of 1.5 s between experiments and the following variable delays: 20, 100, 200, 500, 800, and 1200 ms. Heteronuclear steady-state $^{15}N\{^{1}H\}$ NOE (hetNOE) spectra were acquired with two different data sets, one recorded without an initial proton saturation and the other with an initial proton saturation period of 3 s. The experiments were analyzed with a combination of in-house Mathematica notebooks and CCPNMR software[1].

Constant-time ^{15}N CPMG-TROSY experiments were acquired using [^{15}N,^{2}H]-labeled p38α. The experiments were recorded in an interleaved manner using the following CPMG frequencies: 0, 2000, 25, 50, 1500, 100, 1000, 250, 500, 750, 1250, 25, 100, 250 Hz. We used three repetition frequencies for error calculation, as well as a CPMG delay of total 0.022 s and a recycle delay of 1.5 seconds. The experiments were analyzed with NESSY software[2].

Supplementary figures

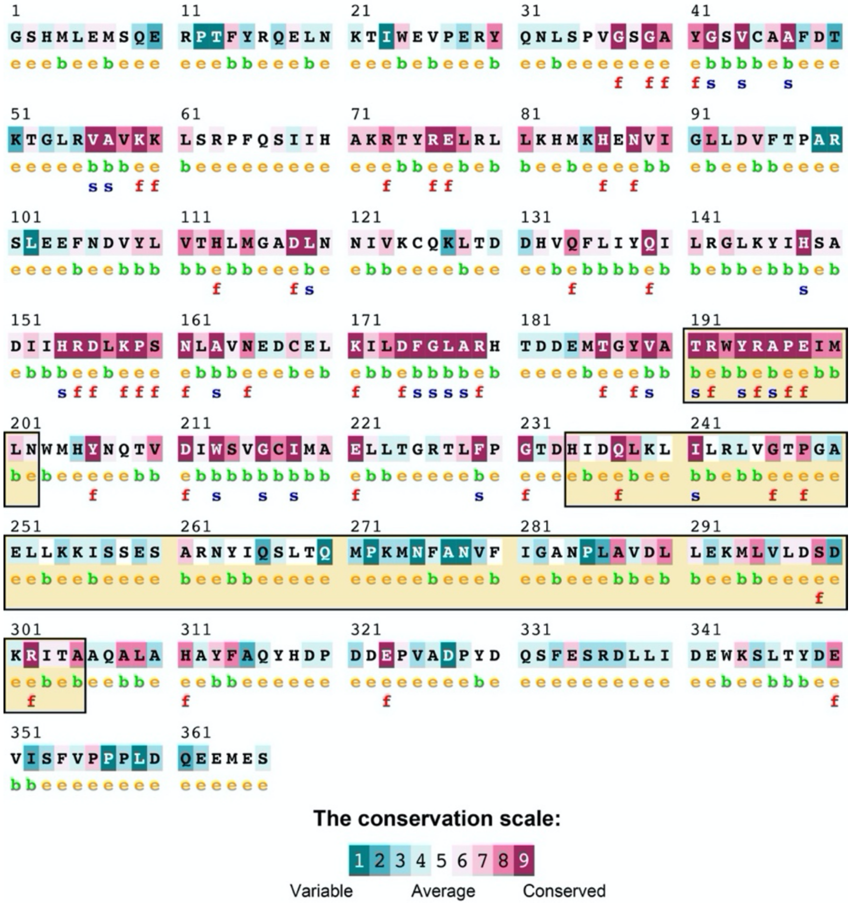

Fig. S1: Sequence conservation profile of p38α kinase generated with ConSurf[3]. The amino acid sequence is colored according to the conservation scale shown at the bottom. The bottom legends indicate; *e* = exposed residue, *b* = buried residue (as predicted by the ConSurf neural network algorithm), *f* = predicted functional residue (highly conserved and exposed), and *s* = predicted structural residue (highly conserved and buried). Residue numbering is shifted by -5 relative to the canonical p38α sequence. Residues part of the lipidic pocket are outlined by black boxes.

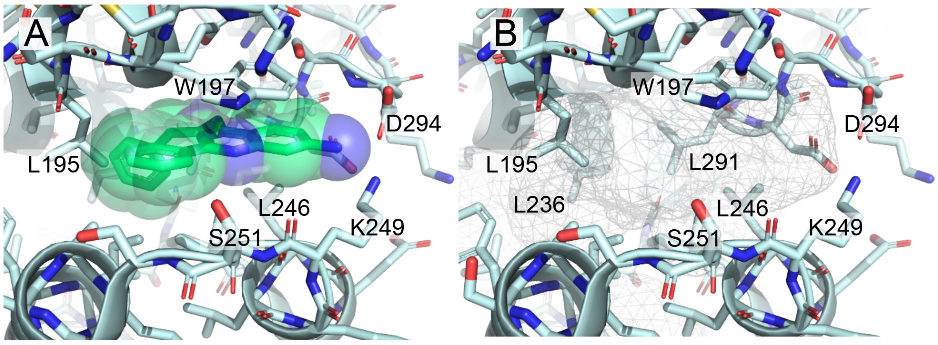

Fig. S2: Zoom-in to the lipidic pocket occupied by an inhibitor. **A)** Depiction of the inhibitor, **B)** the inhibitor being taken out. Interacting residues are annotated. Figure based on PDB 4DLJ.

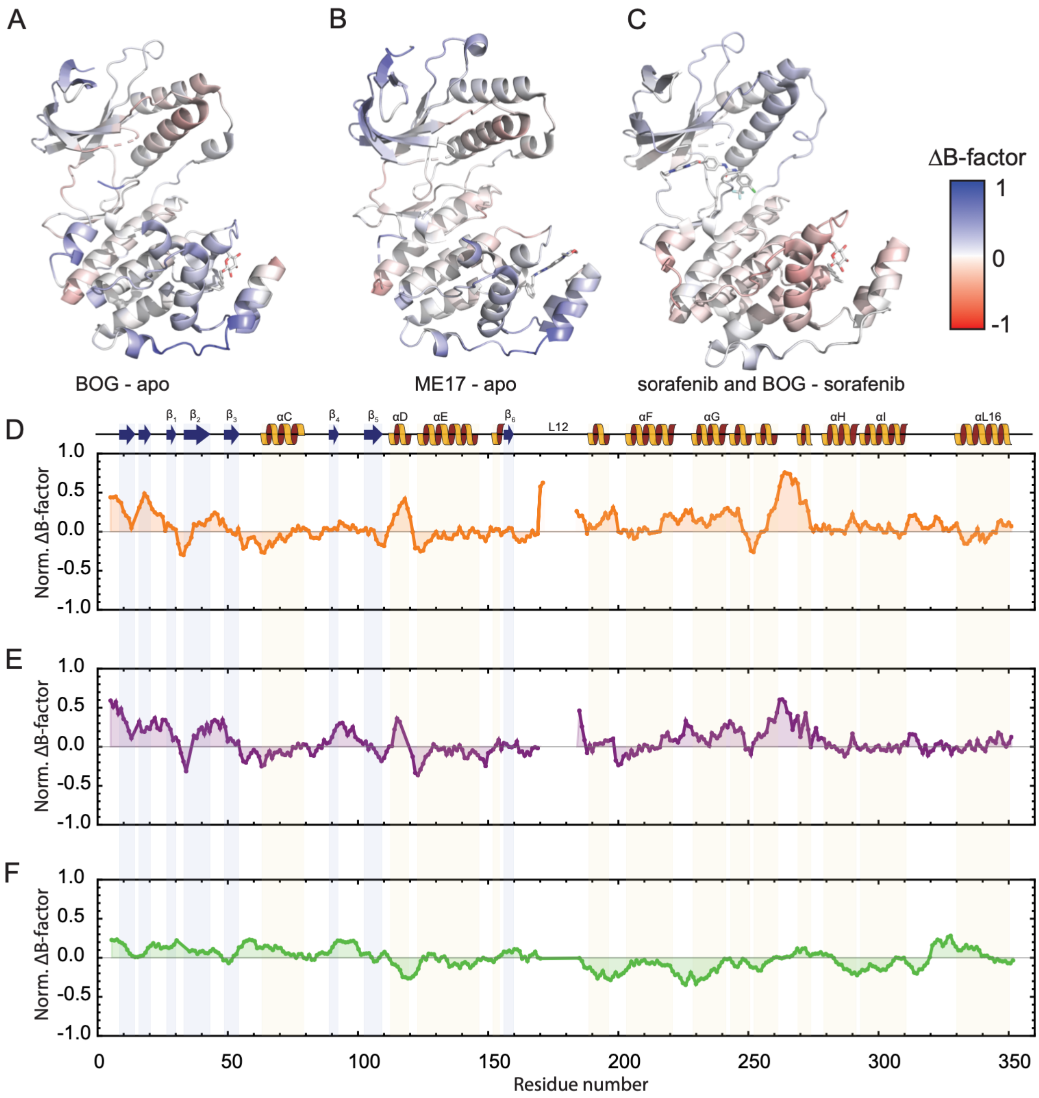

Fig S3: Ligand-induced changes in crystallographic B-factors mapped onto the structure and sequence of p38α. **A-C**) Differences in normalized B-factors (ΔB-factor) between ligand-bound and references states mapped onto the p38α X-ray structure: **A**) BOG-bound minus apo (PDB: 3MH3), **B**) ME17-bound minus apo (PDB: 5N68), and **C**) sorafenib + BOG minus sorafenib-bound p38α (PDB: 3GCS). Color coding reflects the magnitude and sign of the normalized ΔB-factors, as indicated by the scale bar. **D-F**) Corresponding residue-wise profiles of normalized ΔB-factors plotted as a function of sequence position for the same comparison shown in **A-C**, respectively. Secondary-structure elements are indicated above the plots. Normalization (done to compensate for the different absolute scaling of B-factors due to different experimental conditions) refers to dividing each B-factor by the highest value found across each structure before subtracting.

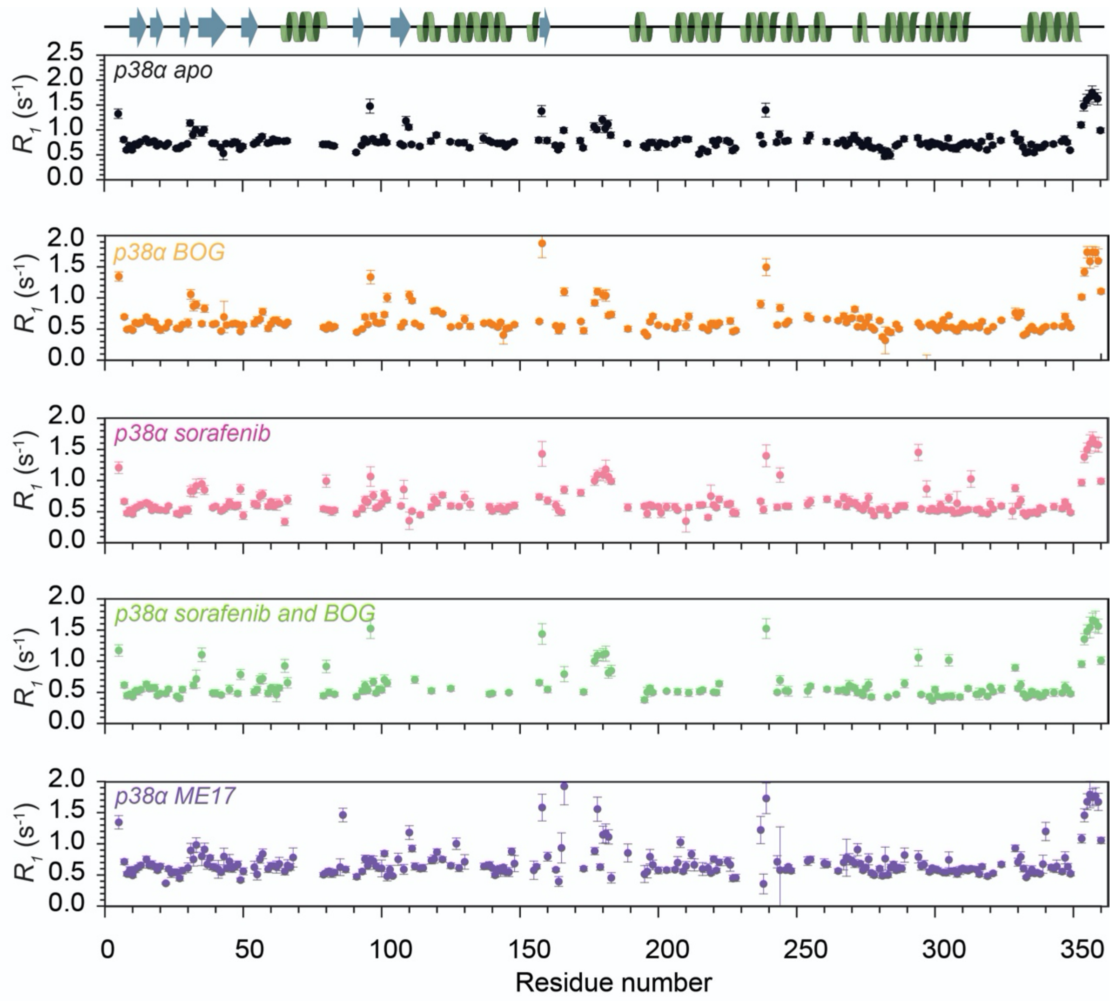

Fig. S4: Comparison of ^{15}N longitudinal R_1 relaxation rates for p38α in different ligand-bound states. Error bars correspond to 1σ standard errors from single-exponential least-squares fits.

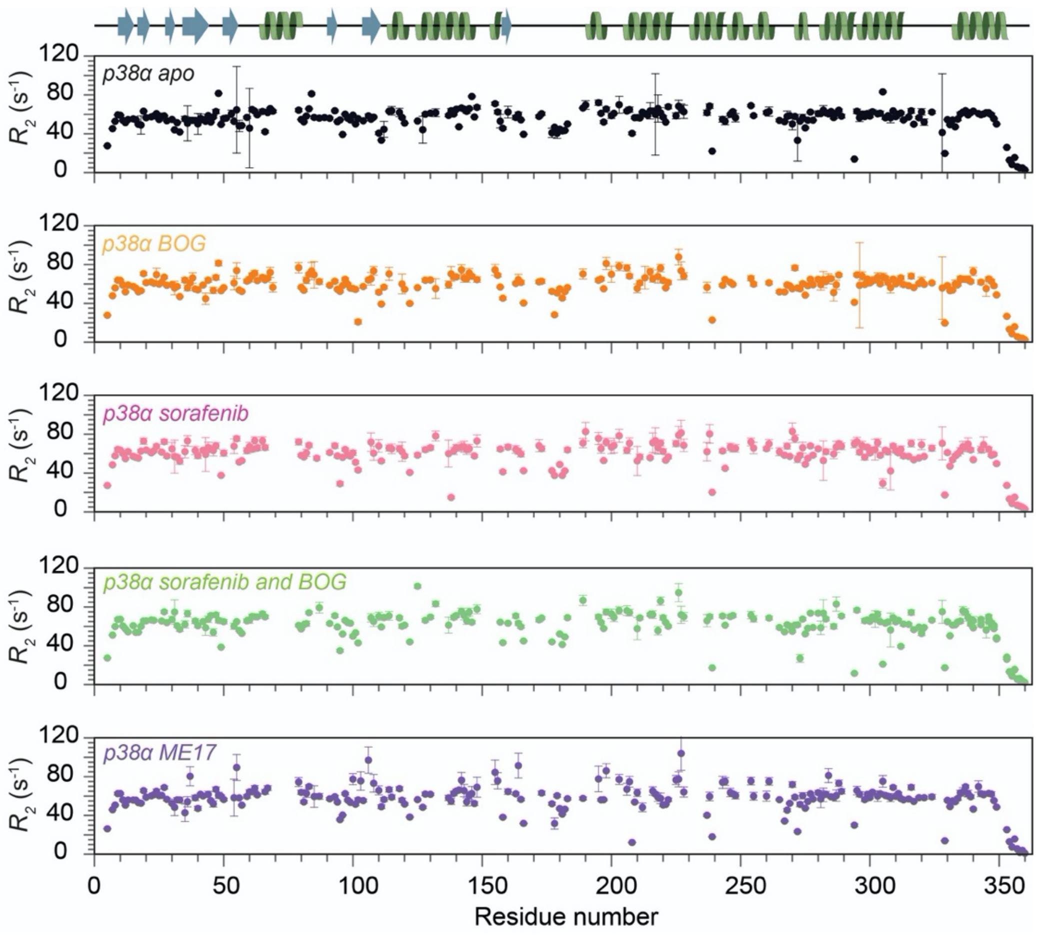

Fig. S5: Comparison of ^{15}N transverse R_2 relaxation rates for p38α in different ligand-bound states. Error bars correspond to 1σ standard errors from single-exponential least-squares fits.

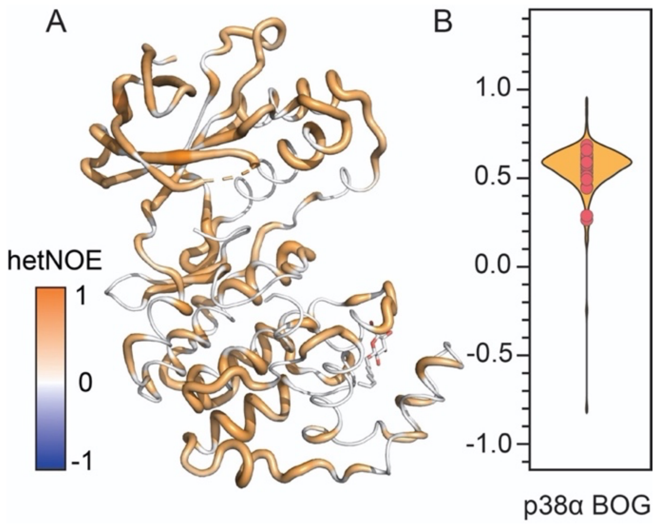

Fig. S6: Distribution of heteronuclear NOE with BOG being bound, represented as **A**) on the protein structure (PDB: 3MH3) and **B**) as a violine plot. Active-site residues in the N-lobe are depicted in red.

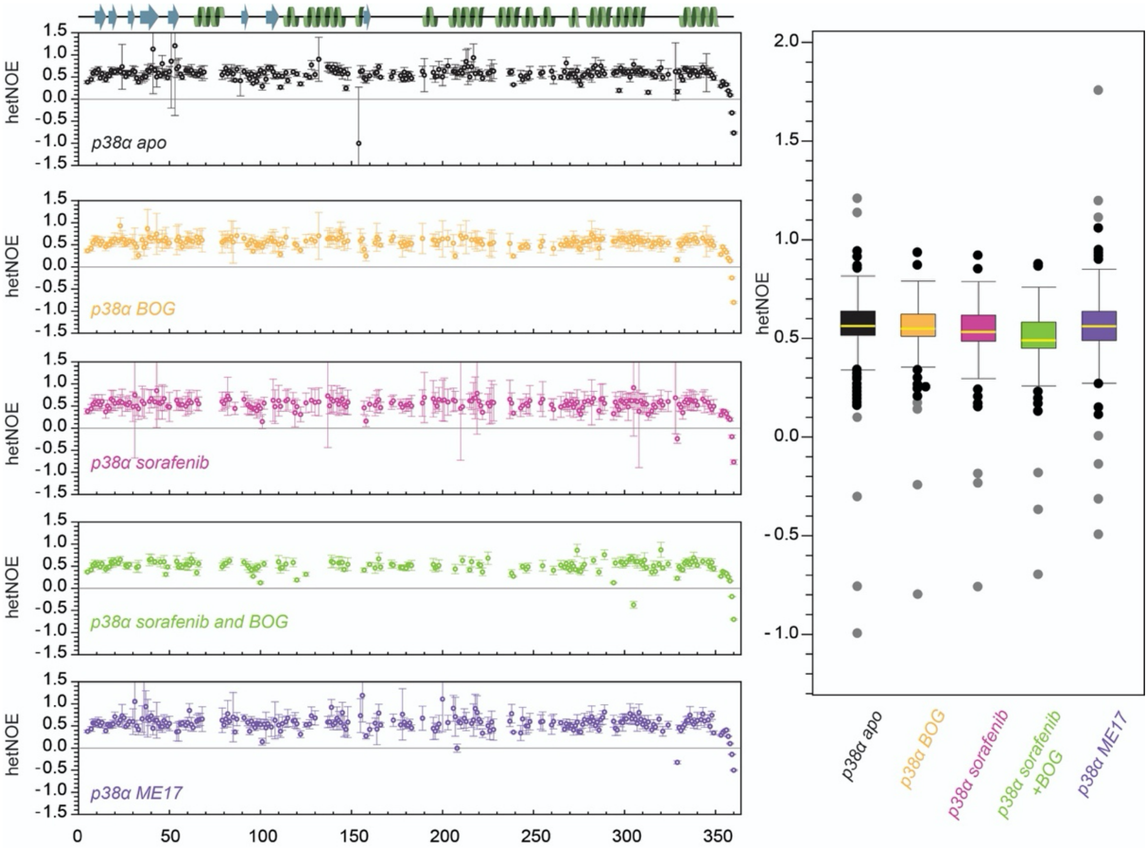

Fig. S7: Comparison of steady-state {^{1}H}-^{15}N heteronuclear NOE values for p38α in different ligand-bound states. The residue-resolved hetNOE profiles are plotted along the sequence (left) and the distributions of hetNOE values for each condition are depicted as box-and-whisker plots with outliers shown on the right. Error bars correspond to uncertainties estimated from peal signal-to-noise ratios and propagated to the NOE values.

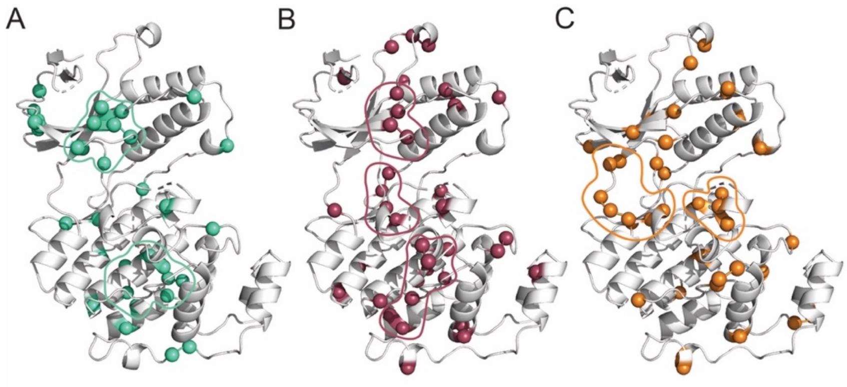

Fig. 8: Mapping of residues exhibiting significant ligand-induced changes in site-specific ^{15}N relaxation parameters onto the apo p38α structure. Residues showing significant differences between the apo protein and ligand-bound states are highlighted for **A**) hetNOE, **B**) R_1, and **C**) R_2 relaxation rates. Differences were calculated as Δ(apo-ligand-bound), and residues exceeding two standard deviations of the respective Δ distributions were classified as significant. The selected residues are mapped onto the apo p38α structures (PDB: 1WFC). Regions with highest spatial densities of significant changes, forming spatial pseudo-clusters, are indicated in each panel.

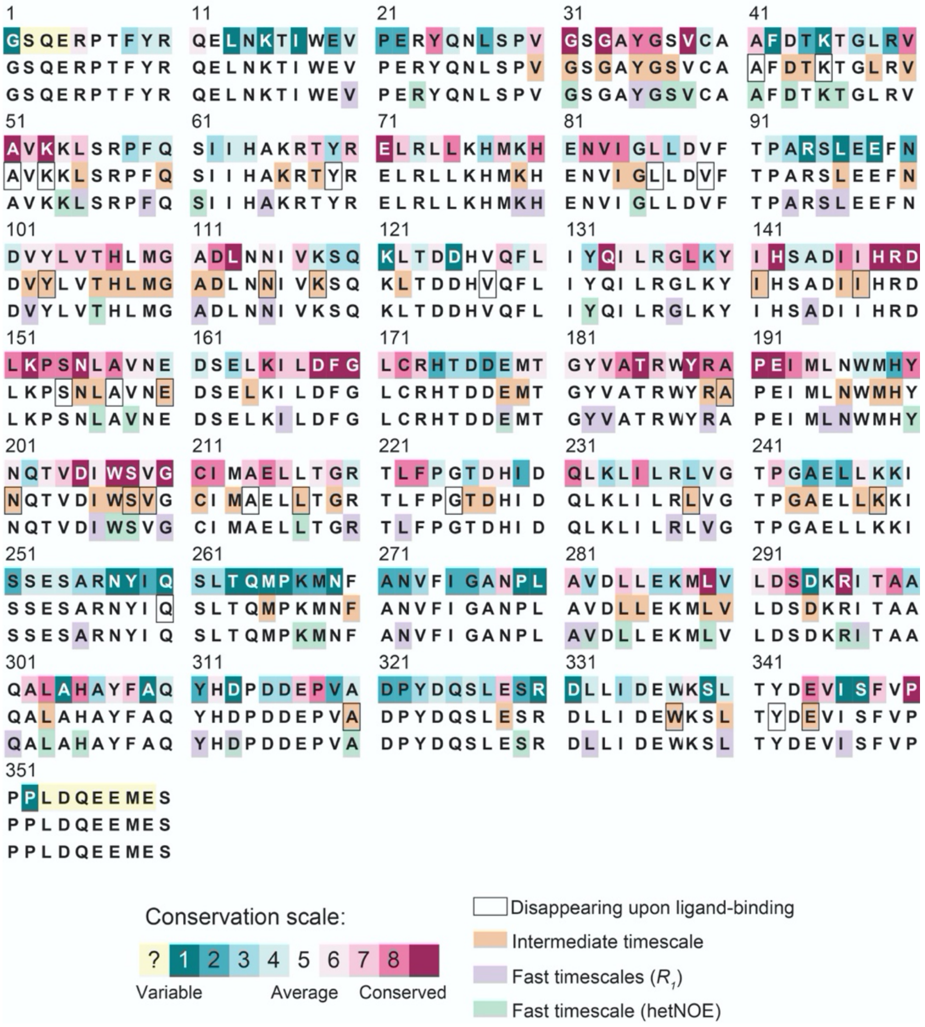

Fig. S9: Sequence conservation in comparison to dynamic signatures of p38α mapped onto the primary structure. The top row shows sequence conservation scores calculated with ConSurf (as in Fig. S1). The middle row highlights residues exhibiting significant ligand-induced deviations associated with intermediate timescale dynamics, as identified from CSPs, CPMG-derived R_{ex}, and R_2 analyses. The bottom row indicates residues affected on fast timescales, based on significant differences in site-specific ^{15}N relaxation parameters, distinguishing contributions observed in R_1 and heteronuclear NOE measurements. Residues were classified as significant using a threshold of two standard deviations relative to the respective experimental distributions.

Citations

[1] S. P. Skinner, R. H. Fogh, W. Boucher, T. J. Ragan, L. G. Mureddu, G. W. Vuister, *J. Biomol. NMR* **2016**, *66*, 111-124.

[2] M. Bieri, P. R. Gooley, *BMC Bioinformatics* **2011**, *12*, 421.

[3] a) M. Landau, I. Mayrose, Y. Rosenberg, F. Glaser, E. Martz, T. Pupko, N. Ben-Tal, *Nucleic Acids Res.* **2005**, *33*, W299-302; b) F. Glaser, T. Pupko, I. Paz, R. E. Bell, D. Bechor-Shental, E. Martz, N. Ben-Tal, *Bioinformatics* **2003**, *19*, 163-164.
